# Determinants of hypertension among diabetic patients in southern Ethiopia: a case-control study

**DOI:** 10.1186/s12872-023-03245-4

**Published:** 2023-05-03

**Authors:** Eyosiyas Abreham Anjajo, Shimelash Bitew Workie, Zegeye Gelan Tema, Beshada Zerfu Woldegeorgis, Efa Ambaw Bogino

**Affiliations:** 1 Department of Internal Medicine, School of Medicine, College of Health Sciences and Medicine, Wolaita Sodo University, Po.box 138, Sodo, Ethiopia; 2 Department of Epidemiology and Biostatistics, School of Public Health, College of Health Sciences and Medicine, Wolaita Sodo University, Po.box 138, Sodo, Ethiopia; 3 School of Medicine, College of Health Sciences and Medicine, Wolaita Sodo University, Po.box 138, Sodo, Ethiopia

**Keywords:** Blood pressure, Cardiovascular disease, Diabetes mellitus, Ethiopia, Hypertension

## Abstract

**Background:**

Hypertension, among diabetic patients, is a worldwide public-health challenge and a number one modifiable risk factor for other cardiovascular diseases and death. The prevalence of hypertension among the diabetic population is nearly twice of nondiabetic patients. Screening and prevention of risk factors for hypertension based on evidence from local studies is required to minimize the burden of hypertension among diabetic patients. This study is aimed at assessing the determinants of hypertension among diabetic patients in Wolaita Sodo University Comprehensive Specialized Hospital, Southern Ethiopia, 2022.

**Methods:**

Facility-based unmatched case-control study design was conducted from March 15 to April 15, 2022, at the outpatient diabetic clinic, Wolaita Sodo University Comprehensive Specialized Hospital. A total of 345 diabetic patients were selected using systematic random sampling techniques. Data were collected using a structured questionnaire by interviewing and extracting from the medical chart of patients. Bivariate logistic regression followed by multiple logistic analysis was used to identify the determinants of hypertension among diabetic patients. A p-value less than 0.05 is considered to be statistically significant.

**Results:**

The significant determinants of hypertension among diabetes patients were being overweight [AOR = 2.06, 95% CI (1.1, 3.89), P = 0.025], being obese [AOR = 2.64, 95% CI (1.22, 5.70), P = 0.013], lack of Moderate intensity exercise [AOR = 2.41, 95% CI (1.36,4.24), P = 0.002], age [AOR = 1.03, 95% CI (1.01, 1.06), P = 0.011], Type 2 diabetes mellitus [AOR = 5.05, 95% CI (1.28, 19.88), P = 0.021], duration of diabetes mellitus ≥ 6 years [AOR = 7.47, 95% CI (2.02, 27.57), P = 0.003], diabetic nephropathy [AOR = 3.87, 95% CI (1.13, 13.29), P = 0.032], and urban residence [AOR = 2.11, 95% CI (1.04,4.29), P = 0.04].

**Conclusion:**

Being overweight and obese, lack of moderate-intensity exercise, age, type 2 diabetes mellitus, duration of Diabetes ≥ 6 years, presence of diabetic nephropathy, and being urban residents were significant determinants of hypertension among diabetic patients. These risk factors can be targeted by health professionals for prevention and earlier detection of hypertension among diabetic patients.

**Supplementary Information:**

The online version contains supplementary material available at 10.1186/s12872-023-03245-4.

## Background

Hypertension, defined as a sustained systolic and diastolic blood pressure greater than 140/90 Millimeters of mercury (mmHg), is common among both type 1 and type 2 diabetes [1]. This definition is predicated on unambiguous data that levels above this threshold are strongly related to mortality and morbidity due to Atherosclerotic Cardiovascular Disease (ASCVD), and microvascular complications as well as antihypertensive treatment in populations with baseline systolic and diastolic blood pressure above this range reduces the occurrence of ASCVD events and microvascular complications. The “sustained” aspect of the hypertension definition is vital, as blood pressure measurements have a considerable normal variation [2].

Atherosclerotic Cardiovascular Disease (ASCVD) is defined as an acute coronary syndrome, myocardial infarction (MI), angina, coronary or other arterial revascularization, stroke, transient ischemic attack, or peripheral arterial disease presumed to be of atherosclerotic origin is the number one cause for morbidity and mortality of people with diabetes and is that the largest contributor to the direct and indirect costs of diabetes. Microvascular complications include diabetic nephropathy, retinopathy, and neuropathy [3, 4].

Diabetes mellitus is a chronic medical condition that is characterized by raised blood glucose levels as a result of very little or no insulin hormone production or insulin resistance in the body. There are three main types of diabetes namely type 1, type 2, and gestational diabetes mellitus. Type 1 diabetes mostly causes diabetes in children but can occur at any age. Type 2 diabetes accounts for the majority which is around 90% of diabetes worldwide. Gestational diabetes mellitus is a type of diabetes where the diagnosis is first made during pregnancy [5].

Hypertension (HTN) is common among patients with diabetes, with the prevalence differing with type and duration of diabetes, age, sex, race/ethnicity, body mass index, history of glycemic control, and the presence or absence of a renal problem, among other factors [6–8]. In addition, Hypertension among diabetic patients remains a strong modifiable risk factor for atherosclerotic diseases, and microvascular complications. Hence, Blood pressure should be measured at every routine clinical care visit for diabetic patients and risk factors for increased blood pressure should be prevented and controlled accordingly [9].

Hypertension and diabetes mellitus have become major public health problems globally, and both are significant risk factors for Atherosclerotic Cardiovascular Disease (ASCVD). Globally, nearly one billion people have HTN; of these, 76% are in developing countries and it is predicted that by 2025, up to 1.56 billion adults worldwide will be hypertensive [10]. Hypertension causes approximately 7.5 million mortality annually, accounting for 57 million disability-adjusted life years, and for about 6% of mortality worldwide [11, 12]. Additionally, diabetes mellitus (DM) is growing at the fastest proportion globally, with the number of adult diabetes population increased more than three times over the past 20 years. The global estimate of the adult diabetes population was 151 million in 2000. By 2009 it had increased by 88% to 285 million. In 2019, International Diabetes Federation calculated that 9.3% of adults aged 20–79 years which is around 463 million people are living with diabetes. Ethiopia is one of the four African countries with the highest number of diabetes (1.7 million) in adults 20–79 years of age [5].

Hypertension, among diabetic patients, is a worldwide public health problem [13]. The diabetic population is nearly twice more affected by hypertension as non-diabetic patients [14]. Compared with other cardiovascular disorders, HTN is the leading comorbid disease in diabetic patients and its effects are devastating if not controlled [15, 16]. Different studies in Africa have shown a high prevalence of Hypertension among diabetic patients. A study conducted in Kenya reported that 50% of diabetic patients had HTN [17]. Another study in Nigeria reported that 54.2% of diabetic individuals had HTN [18]. Similarly, According to the studies conducted in Ethiopia, the prevalence of hypertension among diabetic patients was 59.5% (Debre Tabor) [19], 56.3% (Adama) [20], and 46.5%(Jimma) [21].

The occurrence of both hypertension (HTN) and diabetes mellitus (DM) significantly increases the risk of developing macrovascular complications, leading to a higher incidence of coronary heart disease, heart failure, peripheral arterial disease, and stroke, and also increases the risk of microvascular complications, such as nephropathy or retinopathy [22].In addition, the development of HTN in diabetic individuals complicates the treatment plan and increases the costs associated with health care [23].

Up to 80% of people with diabetes will die of cardiovascular disease, especially HTN and stroke [24, 25]. The presence of both hypertension and DM contributes to the risk of death and cardiovascular events by 44% and 41%, respectively, as compared to 7% and 9% of these risks in people with diabetes alone [26].

The United Kingdom Prospective Diabetes Study demonstrated that blood pressure control results in the prevention of cardiovascular complications in patients with diabetes. Each 10 mmHg decrease in mean systolic blood pressure brings about a 12% reduction of any complication related to diabetes and a 15% reduction in mortality related to diabetes [27]. Older age, overweight/obesity, unhealthy diet, lack of physical exercise, smoking, family history of hypertension, and being an urban resident are major risk factors for HTN [28–31].

The coexistence of hypertension and diabetes has not been given adequate attention in many low-resource setting countries like Ethiopia. In some parts, this is mainly due to health care being stretched by other priorities like Human Immune deficiency Virus/Acquired Immune Deficiency Syndrome (HIV/AIDS), tuberculosis, and malaria. However, the urbanization of the country has created a change within the lifestyles of the population related to nutrition, physical activities, and behaviors like smoking, alcohol, and drug use among urban dwellers, which increases the likelihood of developing non-communicable diseases (NCDs) like hypertension and diabetes. Therefore, it’s time to deal with NCDs particularly the coexistence of hypertension and diabetes.

Moreover, Hypertension is the most important modifiable risk factor for coronary heart disease, stroke, congestive heart failure, end-stage renal disease, and peripheral vascular disease. These risks are more prevalent and serious in diabetic patients. Many Sub-Saharan African countries, including Ethiopia, lack detailed basic data on the determinants of hypertension among diabetic patients [32]. There are few studies on the prevalence of hypertension among diabetes patients, however, published information on determinants of hypertension among diabetes patients is sparse. To date, there is a lack of published evidence regarding the determinants of hypertension among diabetic patients in the Wolaita Zone. Hence, this study aimed at assessing the determinants of hypertension among diabetic patients in Wolaita Sodo University Comprehensive Specialized Hospital, southern Ethiopia.

## Methods

### Study setting and period

The study was conducted from March 15 to April 15, 2022, at Wolaita Sodo University Comprehensive Specialized Hospital which is located in Sodo town, Wolaita Zone, Southern Ethiopia, 329 km from Addis Ababa, the capital city of Ethiopia [33].

### Study design

Facility-based Unmatched case-control study design was conducted.

### Study population

All diabetic patients who were attending the diabetic clinic at Wolaita Sodo University Comprehensive Specialized Hospital for follow-up during the study period were involved in this study. The cases are all diabetic patients diagnosed with hypertension and the controls are all diabetic patients without hypertension who meet the eligibility criteria.

#### Eligibility criteria

##### Inclusion criteria

All diabetic patients aged ≥ 18 years and who had been following up at a diabetic clinic during the study period were included in the study.

##### Exclusion criteria

Patients who were severely ill, pregnant women, not able to communicate, and developed hypertension before diabetes were excluded from the study.

### Sample size determination

The sample size was calculated by using EPI Info software version 7.2.3.1 with the following Parameters: significance = 95%; power = 80%; Adjusted odds ratio (AOR) = 3.9. The Case to control ratio is taken to be 1:2 and The proportion of controls with exposure was 4%. The odds ratio was taken from a study conducted in Debre tabor, Northwest Ethiopia, taking current smoking status as a risk factor for hypertension resulting in the maximum sample size of 311 [19]. Assuming a non-response rate of 10%, the sample size for cases and controls was found to be 115 and 230, respectively, which gave us a total sample of 345.

### Sampling procedure and technique

A systematic random sampling technique was used to select the study subjects. There were 40 patients on average will be seen daily in the diabetic clinic which gives a total of 880 diabetic patients in one-month duration. Two sampling intervals (Ks), one for cases and one for controls, were calculated by dividing the number of cases (250) and controls (630) of the population (N) by their respective number of cases (115) and controls (230) of the sample (n). So, the sampling interval (K) became 2. Therefore, the subjects were selected for every K interval (K = 2) of cases and controls, and the first study subjects were selected by lottery method.

### Study variables

Dependent variables: The presence or absence of hypertension among diabetic patients is the dependent variable.

Independent variables: The independent variables were socio-demographic factors (sex, age, education status, residence, marital status, occupation, ethnicity, and religion), behavioral risk factors (cigarette smoking, alcohol drinking, and chat chewing), health profiles (type and duration of DM, Diabetic self-care practice and family history of HTN and others), and nutritional risk factors (heavy salt consumption, overweight and obesity).

### Data collection tools and procedures

Data was collected by interviewing the study participants and extracting from the medical chart of patients using a structured questionnaire after getting verbal informed consent from the participants. Questionnaires were developed by reviewing different relevant literature. A review of the diabetic patients’ records was conducted to identify cases and controls. Cases and controls were recorded by identification number. Before data collection, the study subjects were identified as cases and controls based on the identification number by the supervisor. The data collectors were blinded to the status of the respondent and they were unable to identify the study subject as case and control.

The questionnaire had three parts: part I, social demographic data and health profile data; part II, Risk factors like behavioral and nutritional factors; and part III, Physical measurement, laboratory tests, and diabetic complications. Laboratory tests were analysed for blood glucose, total cholesterol and HDL cholesterol using CardioCheck PA Analyser and for Triglycerides levels using Cobas Integra 400 Plus (Roche Diagnostics GmbH, Mannheim, Germany) clinical chemistry analyser. The variables that were taken from the medical chart of patients include duration with diabetes since diagnosis, type of diabetes, the presence of complications, and fasting blood sugar during the first diagnosis of diabetic patients of both cases and controls.

### Operational definitions

Hypertension - the average of casual systolic blood pressure readings ≥ 140 mmHg and/or diastolic pressure readings ≥ 90 mmHg [1].

Body Mass Index (BMI) - Underweight: BMI < 18.5 kg/m2; Normal weight: 18.5 ≤ BMI < 25; Overweight: 25 ≤ BMI < 30; obesity BMI ≥ 30 [34].

Waist Circumference: High risk ≥ 94 cm in men and 80 cm in women; substantially high risk > 102 cm in men and > 88 cm in women [35].

Waist to Hip ratio (WHR): Substantially high ≥ 0.90 cm in men and ≥ 0.86 cm in women [35].

Waist-to-height ratio (WHtR): Values for both sexes: low risk 0.40–0.49, high risk 0.50–0.59, and substantially high risk ≥ 0.60 [36].

Glycemic control: Glycemic status was considered as good glycemic control if an average of four consecutive fasting blood glucose measurements 80–130 mg/dL and poor glycemic control if an average of blood glucose values on four consecutive visits were > 130 or < 80 mg/dL [37].

Diabetes Self-care practice: It is a daily activity that the individual patients were performed to manage diabetes on their behalf (dietary practice, exercise, medication, daily foot care, monitoring blood glucose). Diabetes self-care practice was assessed by participants’ responses to the 15-item Summary of Diabetes Self-Care Activities (SDSCA) in the last 7 days. Response choices for each question were range from 0 to 7 based on the number of days on which the indicated behavior was performed. The overall mean score was estimated by the summation of each item of the scale and divided by the total number of questions. Therefore, after calculating the overall mean score, participants who scored equal to or greater than the mean score were classified as having good diabetes self-care practice and those who scored below the mean were considered as having poor self-care practice [38].

Regular exercise: defined in this study as moderate-intensity aerobic physical activity(walking and running) for at least 30 min at least 5 days a week or at least 150 min/per week [39].

Excess salt consumption: people who reported every use of top-added salt on a plate [40].

Heavy Alcohol Consumption - Refers to the average consumption of more than 5 standard alcoholic drinks per day for men (≈ 50gm of alcohol) or > 4 alcoholic drinks (or 40gm of alcohol) for women. A standard alcoholic drink is the equivalent of one glass/can/bottle (330ml) of regular beer (with 3% ethanol), one glass (100ml) of wine (10% ethanol), or one glass or measure (40ml) of distilled spirit, each of which adds up to about 10 g of ethanol per drink [41].

Current Smokers: A person who smokes any quantity of cigarettes in the last 12 months was labeled as a current smoker [42].

Khat chewing: Khat chewer was defined as khat chewing at least once a week while occasional use was defined as khat chewing less than once a week [43].

### Data quality management

The questionnaire was initially prepared in English, and it was translated into Amharic. This questionnaire, prepared in Amharic, was translated back to English to ensure consistency. Data were collected by three nurses (B.Sc.) and two supervisors (B.Sc. /M.Sc.). The training was given to data collectors by the principal investigator and supervisors.

The questionnaire was pre-tested on 5% of the total sample size a week before the actual data collection in Dubo Hospital which is located in Areka town, Wolaita Zone, Southern Ethiopia. Furthermore, the principal investigator and supervisors gave feedback and corrections on daily basis to the data collectors. Completion, accuracy, consistency, and clarity of the collected data were checked regularly.

Weight (in kilograms) was measured in light clothing and without shoes using calibrated standard beam balance that is used for weight measurement in the medical setup. The scale pointer was checked at zero before taking every measurement. The weight of the study participant was measured to the nearest 0.1 kg using a standing beam balance. Height was measured using Stadiometer in centimeters (cm) in an erect position in which the back of the head, shoulder blades, buttocks, and heels make contact with the backboard at a precision. The measurement was recorded to the nearest 0.1 cm. Body mass index was calculated as weight in kilograms divided by the square of height in meters (kg/m2) [44].

Waist and hip circumferences were measured using a non-stretch tape meter with 0.1 cm precision. Waist circumference was measured by placing a tape measure around the bare abdomen at the midpoint between the lower margin of the last palpable rib and the top of the iliac crest of the hip bone. Hip circumference was measured by placing a tape measure around the hip at the maximum circumference over the buttocks or around the greater trochanter of the femoral bone. The waist-to-hip ratio (WHR) and Waist to height ratio (WHtR) were calculated using measurements of waist, hip circumferences, and height [45].

### Data processing and analysis

The collected data were entered into Epi data version 4.6.0.2 and analyzed using Statistical Package for Social Science (SPSS) version 25. Percentages and frequencies were used to summarize categorical variables. The results were presented by tables and graphs based on the nature of the variable. The distribution of the continuous variables was checked for normality distribution. Mean with standard deviation and median with interquartile range were used to summarize normally and non-normally distributed continuous variables respectively.

Analysis using a bivariate logistic regression model was used to see the association between the explanatory variables and the outcome variable. This was followed by multivariable logistic regression analysis using those variables with a P-value of 0.2 or less in the bivariable analysis. To check the goodness of fit of the statistical model, the Hosmer-Lemeshow test was used. Multicollinearity was assessed by the Tolerance test and variance inflation factor. The Odds ratio with 95% CI was used to measure the strength between the dependent and the independent variables. A p-value less than 0.05 was used to determine the level of statistical significance.

## Results

### Socio-demographic characteristics of the respondents

A total of 115 diabetic patients who had hypertension (cases) and 230 DM patients who had no hypertension (controls) were included with a response rate of 100%. The mean (± SD) age was 55.1 (± 11.9) years for cases and 44.3 (± 13.8) years for controls. Forty-eight cases (41.7%) and 85 (37.0%) controls were female participants. Most of the cases (85.2%) and controls (61.3%) were living in urban areas. Most of the cases (62%) and controls (70%) attended only secondary education or less. In addition, most of the cases (98.3%) and controls (87%) were married. While 30% (30.4%) of the cases were housewives then followed by government employees and private work, only 24% (24.3%) of the control were in private work then followed by government employees and housewives (Table 1).

**Table 1:** Socio-demographic characteristics of people with diabetes on follow-up at Wolaita Sodo University Comprehensive Specialized Hospital, Southern Ethiopia, 2022

Variables		Cases, no. (%)	Controls, no. (%)	P value
Sex	Female	67(58.3%)	145(63%)	0.39
	Male	48(41.7%)	85(37%)	
Residence	Urban	98(85.2%)	141(61.3%)	0.0001
	Rural	17(14.8%)	89(38.7%)	
Marital status	Single	2(1.7%)	29(12.6%)	0.305
	Married	113(98.3%)	200(87%)	
Educational status	No education	19(16.7%)	38(16.5%)	
	Primary	25(21.9%)	61(26.5%)	0.589
	Secondary	26(22.8%)	60(26.1%)	0.696
	More than Secondary	44(38.6%)	71(30.9%)	0.528
Occupation	Private work	19(16.5%)	56(24.3%)	
	Government employee	31(27%)	49(21.3%)	0.376
	Farmer	6(5.2%)	37(16.1%)	0.241
	Housewife	35(30.4%)	45(19.6%)	0.217
	Retired	23(20%)	16(7%)	0.252
	Unemployed	1(0.9%)	27(11.7%)	0.335
Monthly income	</ = 1000 ETB	39(34.2%)	73(35.6%)	
	1001–5000 ETB	60(52.6%)	111(54.1%)	0.963
	> 5000 ETB	15(13.2%)	21(10.2%)	0.459

### Health profile of the respondents

The median (IQR) reported duration of DM from the diagnosis was 8 [5, 14] years in cases and 5 [2, 10] years in controls. 15% of the cases and 5.2% of the controls reported a family history of HTN. The majority of the cases (97.4%) and controls (72.2%) were having type two DM. Of the study participants, 44.3% of the cases and 48.7% of the controls reported using insulin. The majority of cases (41.7%) but only a few of the control (23%) were overweight and a higher percentage of cases (27.8) as compared to controls (13%) were obese (Table 2).

**Table 2:** Health profile of people with diabetes on follow-up at Wolaita Sodo University Comprehensive Specialized Hospital, Southern Ethiopia, 2022

Variables		Cases, no. (%)	Controls, no. (%)	P value
Type of DM	type one	3(2.6%)	64(27.8%)	0.0001
	type two	112(97.4%)	166(72.2%)	
DM duration	≤ 1 year	3(2.6%)	40(17.5%)	
	2–5 year	30(26.1%)	79(34.6%)	0.011
	≥6 year	82(71.3%)	109(47.8%)	0.000
Family history of hypertension	Yes	18(15.7%)	12(5.2%)	0.002
	No	97(84.3%)	218(94.8%)	
Family history of Diabetes	Yes	25(22.1%)	35(15.4%)	0.128
	No	88(77.9%)	192(84.6%)	
Attended Diabetic education	Yes	12(10.4%)	30(13%)	0.486
	No	103(89.6%)	200(87%)	
Member of the diabetes association	Yes	15(13%)	23(10%)	0.396
	No	100(87%)	207(90%)	
Have a glucometer at home	Yes	17(14.8%)	29(12.6%)	0.576
	No	98(85.2%)	201(87.4%)	
Comorbidity	Yes	10(8.7%)	12(5.2%)	0.22
	No	105(91.3%)	218(94.8%)	
BMI status	< 25 kg/m2	35(30.4%)	147(64%)	
	25–29.9 kg/m2 (Overweight)	48(41.7%)	53(23%)	0.0001
	≥ 30 kg/m2 (Obese)	32(27.8%)	30(13%)	0.0001
Diabetes self-care practice	Poor	64(55.7%)	145(63%)	0.286
	Good	51(44.3%)	85(37%)	
Ever smoked tobacco products	Yes	5(4.3%)	3(1.3%)	0.095
	No	110(95.7%)	227(98.7%)	
Ever drunk alcoholic drinks	Yes	13(11.3%)	19(8.3%)	0.360
	No	102(88.7%)	211(91.7%)	
Top added salt	Yes	33(28.7%)	64(27.8%)	0.87
	No	82(71.3%)	166(72.2%)	
Vigorous intensity exercise	Yes	16(13.9%)	33(14.3%)	0.91
	No	99(86.1%)	197(85.7%)	
Moderate intensity exercise	Yes	52(45.6%)	159(69.4%)	0.0001
	No	62(54.4%)	70(30.6%)	
Diabetic nephropathy	Yes	17(14.8%)	6(2.6%)	0.0001
	No	98(85.2%)	224(97.4%)	
Diabetic retinopathy	Yes	40(34.8%)	49(21.3%)	0.007
	No	75(65.2%)	181(78.7%)	
Glycemic control	Good	16(14%)	36(16%)	0.636
	Poor	98(86%)	189(84%)	

### Metabolic risk factors of hypertension

The median SBP (Q1, Q3) of the cases were 146 (136,155) mmHg in cases and 126 (121,134) mmHg in controls. Then, the median DBP (Q1, Q3) of the cases was 85 (79, 92) mmHg in cases and 79 (73, 85) mmHg in controls **(**Table 3**)**.

**Table 3:** Metabolic factors of people with diabetes on follow-up at Wolaita Sodo University Comprehensive Specialized Hospital, Southern Ethiopia, 2022

Variables		Cases		Controls	
		Median	IQR(Q1,Q3)	Median	IQR(Q1,Q3)
Mean FBS in mg/dl(n = 339)		166	74(136,210)	182	88(144,232)
Hemoglobin A1c % (n = 37)		6.4	1.2(5.9,7.1)	7.2	1.9(6.2,8.1)
Triglyceride in mg/dl (n = 132)		140	50(109,159)	142	80(110,190)
Total cholesterol in mg/dl (n = 138)		180	74(146,220)	154	55(135,190)
HDL cholesterol in mg/dl (n = 83)		46	18(34,52)	40	22(30,52)
LDL cholesterol in mg/dl (n = 90)		108	59(73,132)	108	70(63,133)
Creatinine in mg/dl (n = 208)		1	0.4(0.8,1.2)	0.9	0.4(0.7,1.1)
Mean SBP in mmHg(n = 345)		146	19(136,155)	126	13(121,134)
Mean DBP in mmHg (n = 345 )		85	13(79,92)	79	12(73,85)
Waist Circumference	Male	96	9(92,101)	90	15(82,96.5)
	Female	106	16(98,114)	96	20(85.5,105)
Waist to Hip ratio (WHR)	Male	0.96	0.06(0.94,1.0)	0.95	0.06(0.91,0.98)
	Female	0.97	0.05(0.94,0.99)	0.95	0.07(0.91,0.98)
Waist-to-height ratio (WHtR)	Male	0.57	0.08(0.53,0.60)	0.53	0.09(0.49,0.57)
	Female	0.66	0.1(0.60,0.70)	0.59	0.11(0.54,0.65)

Key: FBS = Fasting blood sugar, LDL = Low density lipoprotein, HDL = High density lipoprotein

### Determinants of hypertension among Diabetic patients

The bivariate logistic regression model was used to see the association between the explanatory variables and the outcome variable. Then, eleven variables were selected for the multivariable logistic regression analysis using the P value less than 0.2 from the bivariate logistic regression. These included Age, Place of residence, type of DM, duration of DM, family history of Hypertension, family history of DM, smoking history, moderate-intensity exercise, BMI, diabetic nephropathy, and diabetic retinopathy. The crude and adjusted odds ratios along with their 95% confidence interval were determined. Then, a P-value less than or equal to 0.05 was considered to be statistically significant.

Multiple logistic regression analysis results identified no significant difference among cases and control for the variables including a family history of hypertension, family history of DM, ever-smoked tobacco products, and diabetic retinopathy in this study.

Based on the result of the multivariable logistic regression analysis, the following variables were found to be independent determinants of hypertension among diabetic individuals at a P value less than 0.05: Age, place of residence, type of DM, Duration of DM, Moderate intensity exercise, BMI, diabetic nephropathy and diabetic retinopathy (Table 4). The model fitness was checked by Hosmer and Lemeshow test (chi-square = 8.843) with P-value = 0.356, and overall 82.7% of variables were correctly classified.

Being overweight and obese were significantly associated with hypertension among diabetic patients. The odds of hypertension were 2.06 times higher among overweight diabetic individuals than those with normal weight [AOR = 2.06, 95% CI (1.1, 3.89), P = 0.025]. Similarly, obese individuals with diabetes had about 2.64 times higher odds of developing hypertension when compared with normal weight [AOR = 2.64, 95% CI (1.22, 5.70), P = 0.013].

Moderate-intensity exercise was significantly associated with lower odds of hypertension among diabetic individuals. Diabetic individuals with a lack of moderate-intensity exercise had about 2.4 times higher odds to develop hypertension [AOR = 2.41, 95% CI (1.36, 4.24), P = 0.002].

Age was another significant independent determinant of hypertension among diabetic patients. For each one-year increase in age, there was a 3% increased odds of developing hypertension among diabetic patients [AOR = 1.03, 95% CI (1.01, 1.06), P = 0.011].

The type and duration of diabetes was also significant determinant of hypertension among the diabetes population. Type 2 DM patients had 5 times higher odds of developing hypertension when compared to type 1 DM patients [AOR = 5.05, 95% CI (1.28, 19.88), P = 0.021]. Similarly, the odds of hypertension were 7 times higher when the duration of DM is longer than 5 years since diagnosis [AOR = 7.47, 95% CI (2.02, 27.57), P = 0.003].

The presence of diabetic nephropathy was another independent risk factor for the development of hypertension in diabetic patients. The odds of hypertension were around four times higher among diabetic nephropathy patients when compared to individuals without diabetic nephropathy [AOR = 3.87, 95% CI (1.13, 13.29), P = 0.032].

Finally, being an urban resident had been found as an independent risk factor for the development of hypertension among diabetic patients. The odds of hypertension among urban residents were two times higher than the rural residents in this study [AOR = 2.11, 95% CI (1.04, 4.29), P = 0.04] (Table 4).

**Table 4:** Multivariable logistic regression analysis results of determinants of hypertension among diabetic people on follow-up at Wolaita Sodo University Comprehensive Specialized Hospital, Southern Ethiopia, 2022

Variables	Cases	Controls	COR (95% CI)	AOR (95% CI)	P- Value
Age (Mean ± SD)	55.1(± 11.9)	44.3(± 13.8)	1.07(1.04,1.09)	1.03(1.01,1.06)*	0.011
Residence					
Urban	98(85.2%)	141(61.3%)	3.64(2.04,6.49)	2.11(1.04,4.29)*	0.04
Rural	17(14.8%)	89(38.7%)	1	1	
Type of DM					
type one	3(2.6%)	64(27.8%)	1	1	
type two	112(97.4%)	166(72.2%)	14.39(4.41,46.95)	5.05(1.28,19.88)*	0.021
DM duration					
≤ 1 year	3(2.6%)	40(17.5%)	1	1	
2–5 year	30(26.1%)	79(34.6%)	5.06(1.46,17.61)	3.73(0.97,14.35)	0.055
≥6 year	82(71.3%)	109(47.8%)	10.03(2.99,33.56)	7.47(2.02,27.57)*	0.003
Family history of HTN					
yes	18(15.7%)	12(5.2%)	3.37(1.56,7.27)	2.10(0.81,5.46)	0.126
No	97(84.3%)	218(94.8%)	1	1	
Family history of DM					
yes	25(22.1%)	35(15.4%)	1.56(0.88,2.76)	1.08(0.51,2.28)	0.840
No	88(77.9%)	192(84.6%)	1	1	
Ever smoked tobacco products					
yes	5(4.3%)	3(1.3%)	3.44 (0.81,14.65)	1.96(0.35,10.88)	0.443
No	110(95.7%)	227(98.7%)	1	1	
Moderate intensity exercise					
yes	52(45.6%)	159(69.4%)	1	1	
No	62(54.4%)	70(30.6%)	2.71(1.70,4.30)	2.41(1.36,4.24)*	0.002
BMI					
< 25 kg/m2	35(30.4%)	147(64%)	1	1	
25–29.9 kg/m2 (Overweight)	48(41.7%)	53(23%)	3.80 (2.22,6.51)	2.06(1.1,3.89)*	0.025
≥ 30 kg/m2 (Obese)	32(27.8%)	30(13%)	4.46(2.41,8.33)	2.64(1.22,5.70)*	0.013
Diabetic nephropathy					
Yes	17(14.8%)	6(2.6%)	6.48 (2.48,16.92)	3.87(1.13,13.29)*	0.032
No	98(85.2%)	224(97.4%)	1	1	
Diabetic retinopathy					
Yes	40(34.8%)	49(21.3%)	1.97 (1.20,3.24)	1.04(0.56,1.94)	0.905
No	75(65.2%)	181(78.7%)	1	1	

* Statistically significant (p-value ≤ 0.05)

## Discussion

This study identified Overweight, Moderate intensity exercise, Age, Type and duration of DM, diabetic nephropathy, and urban dwellers as independent determinants of hypertension among diabetic individuals in Wolaita Sodo University Comprehensive Specialized Hospital, Southern Ethiopia,2022.

Being overweight and obese were significantly associated with hypertension among diabetic patients. The odds of hypertension were 2.06 times higher among overweight diabetic individuals than those with normal weight. Similarly, obese individuals with diabetes had about 2.64 times higher odds of developing hypertension when compared with normal weight. This is consistent with previous studies conducted in Nigeria [46], Iraq [47], and Ethiopia [48, 49]. Several mechanisms relate obesity to hypertension including dietary factors, vascular injury, renal dysfunction, sympathetic over-activation, inappropriate RAAS activation, structural and functional abnormalities in the kidney and heart, insulin resistance, and Immune dysfunction [50].

Moderate-intensity exercise was significantly associated with lower odds of hypertension among diabetic individuals. Diabetic individuals with a lack of moderate-intensity exercise had about 2.4 times higher odds to develop hypertension. This result is consistent with previous studies done in different countries [51–54]. Different epidemiological studies have shown decrement in both systolic and diastolic pressure in the general population with moderate-level intensity exercisers. Moderate-intensity exercise is beneficial and recommended for both the prevention and treatment of hypertension [55]. The proposed mechanisms by which moderate-intensity exercise reduces blood pressure include increased insulin sensitivity, beneficial changes in autonomic nervous system function, and vasoconstriction regulations [51].

Age was another significant independent determinant of hypertension among diabetic patients. For each one-year increase in age, there was a 3% increased odds of developing hypertension among diabetic patients. This finding is similar with studies done in Netherland [56] ,Morocco [57], United Arab Emirates [58], Libya [59] and Ethiopia [60, 61]. When their age increases, individuals will adopt a sedentary lifestyle and gain weight which predisposes them to hypertension. Age is one of the non-modifiable risk factors of hypertension increasing peripheral vascular resistance associated with atherosclerotic changes in the vessels [55].

The type and duration of diabetes were other significant determinant of hypertension among the diabetes population. Type 2 DM patients had 5 times higher odds of developing hypertension when compared to type 1 DM patients. Similarly, the odds of hypertension were 7 times higher when the duration of DM is longer than 6 years since diagnosis. This finding is similar to studies done in different countries [6–8, 30]. Although there is a higher prevalence of hypertension and other cardiovascular diseases among Type 2 DM, Type 1 DM patients are still at higher risk, particularly with a longer duration of DM. This is explained partly by the obesity epidemic, glycemic control, pathology in the arterial wall, abnormal inflammation cascade, and atherosclerosis [6].

The presence of diabetic nephropathy was another independent risk factor for the development of hypertension in diabetic patients. The odds of hypertension were around four times higher among patients with diabetic nephropathy when compared to individuals without diabetic nephropathy. Hypertension is one of the leading comorbidity of patients with CKD. This result is consistent with different studies conducted in different countries [62–64]. The prevalence of hypertension among CKD patients increases with a progressive decline in renal function [65]. Hypertension may develop as a result of kidney disease and the presence of hypertension also worsens further decline of renal function [55]. The mechanism of hypertension in kidney disease is related to decreased capacity of the kidney to excrete sodium, hypersecretion of renin, and increased activation of the sympathetic nervous system [66].

Finally, being an urban resident had been found as an independent risk factor for the development of hypertension among diabetic patients. The odds of hypertension among urban resident was two times higher than the rural residents in this study. This finding is similar to studies done in India [67], Ghana [68], and Ethiopia [19, 31]. urbanization has created a change in the lifestyles of the population related to nutrition, physical activities, and behaviors like smoking, alcohol, and drug use among urban dwellers, which increases the likelihood of developing hypertension [69, 70]. The development of hypertension is influenced by various environmental factors associated with urbanization including dietary factors, physical activity, and alcohol intake [22].

### Strength and limitation of the study

The design of the study used a case-control study to evaluate the determinant of hypertension is one of the strengths of the study. The other strength of the study is the recruitment of the large sample size in the study. The study also included multiple potential risk factors to determine their association with the development of hypertension. The study also utilized different data collection methods including interviews, medical chart review, and measurements.

The limitation of the study includes the fact that arises from the very nature of case-control design which is difficult to determine the temporal association between the outcome and risk factors. It is also a single facility-based study. Random and systemic measurement errors may also affect the result in variables reported by the individual.

## Conclusions

Hypertension, among diabetic patients, is a worldwide public-health challenge and a number one modifiable risk factor for other cardiovascular diseases and death. The prevalence of hypertension among the diabetic population is nearly twice of nondiabetic patients. Hence, Screening and prevention of risk factors for hypertension based on evidence from local studies is required to minimize the burden of hypertension among diabetic patients.

This study has identified Overweight and obesity, Moderate intensity exercise, Age and type and duration of DM, diabetic nephropathy, and urban residence as independent determinants of hypertension among diabetes patients. These risk factors can be targeted for effective screening and prevention of hypertension in diabetic patients.

These research findings suggest screening and prevention of hypertension can be intensified among type two diabetic Patients and diabetic patients with a longer duration of the disease. Moderate-intensity exercise for at least thirty minutes per day for at least five days per week can be prescribed for all diabetic patients. Overweight and obese patients can also be enrolled in weight reduction interventions and given close follow-up to prevent hypertension in these high-risk groups.

Moderate-intensity exercise and prevention of overweight and obesity are also important modifiable risk factors that can be applied on large scale to prevent hypertension in diabetic patients. Large-scale screening and detection programs can be implemented in these high-risk groups to tackle this problem at the community level. Healthcare professionals, public health professionals, and nutrition professionals can expand this recommendation to a large scale to produce a significant result in the prevention and management of hypertension in diabetic patients.

In addition, further Multicenter studies can be studied to investigate risk factors across large sample sizes and different study settings to determine the risk factors for the determinant of hypertension among the diabetic population. This can increase the knowledge of risk factors as well as possible intervention strategies from large-scale studies in different study settings which can be extrapolated to the population level.

## Electronic supplementary material

Below is the link to the electronic supplementary material.

Supplementary Material 1 The questionnaire used for data collection in this study is included in the supplementary file.

## Data Availability

The datasets used and analysed during the current study are available from the corresponding author upon reasonable request. The questionnaire used in this study is included in the Supplementary file.

## Abbreviations

AOR: Adjusted Odds Ratio
ASCVD: Atherosclerotic Cardiovascular Disease
BMI: Body Mass Index
CI: Confidence Interval
COR: Crude Odds Ratio
DM: Diabetes mellitus
HIV/AIDS: Human Immune deficiency Virus/Acquired Immune Deficiency Syndrome
HTN: Hypertension
mmHg: Millimeters of Mercury
MI: Myocardial Infarction
NCDs: Non-Communicable Diseases
SD: Standard deviation
SPSS: Statistical Package for Social Science
WHO: World Health Organization
