# Supplementary material for: Determinants of hypertension among diabetic patients in southern Ethiopia: a case-control study
Source: BMC Cardiovasc Disord. 2023 May 3;23:233. doi: 10.1186/s12872-023-03245-4 (PMC10157915; doi:10.1186/s12872-023-03245-4)
Supplement: Supplementary file 1 — Supplementary Material 1 The questionnaire used for data collection in this study is included in the supplementary file. [file 12872_2023_3245_MOESM1_ESM.docx]

**Questionnaire**

Questionnaire Code Number **____________** Code type 1. DM + HTN 2. DM only

Address: city/Sub city _______ Woreda _______Medical Card Number________ ( **see Chart)

| No | **I. Socio demographic characteristics** | Response classification | | ** |
| --- | --- | --- | --- | --- |
|  | Sex of the respondent | 1. Male 2. Female | |  |
|  | Age ( in years) | ________________________ | |  |
|  | What is your marital status? | 1. Never married 2. Married 3. Separated | 1. Divorced 2. Widowed |  |
|  | Where is your place of residence? | 1. Urban 2. Rural | |  |
|  | What is your highest level of education attended? | 1. No education 2. Primary | 1. Secondary 2. More than Secondary |  |
|  | What is your Religion? | 1. Orthodox 2. Catholic 3. Protestant 4. Muslim 5. Traditional 6. Other (specify)____________ | |  |
|  | What is your current occupation? | 1. Student 2. Government employee 3. Self employed 4. Daily laborer 5. Merchant 6. Housewife 7. Farmer 8. No job 9. Other (specify)____________ | |  |
|  | How much income you earn monthly in average? (Ethiopian Birr) | _____________birr | |  |
|  | Family size? | _____________members | |  |
| Health Profile Questions | | | | |
|  | Type of DM | 1. Type 1  2. Type 2 | |  |
|  | Duration of DM since diagnosis (write in months if less than 1 year)? | ­­­  __________________Years(months) | |  |
|  | Duration since DM treatment started (write in months if less than 1 year)? | __________________Years(months) | |  |
|  | Indicate the **date** of **start** for only those medications you ever used out of the following antidiabetic medications? (DD/MM/YY) | 1. Metformin_____/______/______ 2. Glibenclamide(Daunil)___/____/___ 3. Insulin_____/______/______ 4. Other (specify) ____/____/______ | |  |
|  | How long have you been using the drugs you described above? Please Indicate the **date** you **stopped** taking the medicine for each of the followings that is applicable to you. (DD/MM/YY) | - 1. Metformin_____/______/______   2. Glibenclamide(Daunil) ___/____/___   3. Insulin_____/______/______   4. Other(specify)______/______/_____ | |  |
|  | Have you ever missed any of the doses of anti-diabetic drug that was ordered by your doctor? | 1. Yes → If ‘Yes’ Go to 16 2. No → If ‘No’ Go to 17 | |  |
|  | If yes to question 15, how many doses have you missed in the last month? | _____________doses/month | |  |
|  | On how many of the last SEVEN DAYS, did you take your recommended diabetes medication? Number of days per week | 0 1 2 3 4 5 6 7 | |  |
|  | Were there anytime that you have quitted your anti-diabetic medication? | 1. Yes → If ‘Yes’ Go to 19 2. No → If ‘No’ Go to 20 | |  |
|  | If yes to question no. 18, for how long have you quitted? | _________weeks | |  |
|  | Are you on regular follow up for diabetes? | - - 1. Yes 2.No | |  |
|  | Do you attend diabetic education session? | 1. Yes 2.No | |  |
|  | Are you a member of diabetes association? | 1. Yes 2.No | |  |
|  | Do you have glucometer at home? | 1. Yes 2.No | |  |
|  | Current DM treatment? | 1. Insulin injection  2. Oral medication  3. both  4. I don’t take medication | |  |
|  | Do you have history of diabetic in your family? | 1. Yes 2. No | |  |
|  | **S**ource of information about diabetics and therapy? | 1. Medical staff 2. Media 3. Relatives and friend 4. From diabetic association 5. Others (Specify)_____________ | |  |
|  | Are you taking drug for hypertension? | 1. Yes 2. No | |  |
|  | Have you been diagnosed with hypertension? ** | 1. Yes → If ‘Yes’ Go to 29  2. No → If ‘No’ Go to 30 | |  |
|  | If yes for Q28, How long have you been since diagnosed with hypertension? | ___________year | |  |
|  | Do you have family history of hypertension? | 1. Yes 2.No | |  |
|  | Do you have comorbidity confirmed by physician other than hypertension? Look for medical chart like HIV, TB, CHF etc….** | 1. Yes → If ‘Yes’ Go to 32  2. No → If ‘No’ Go to 33 | |  |
|  | If yes to 31, what is the diagnosis? **  (RECORD ALL with date of diagnosis) |  | |  |
| **Diabetic Self-Care Practices** | | | | |
|  | **Diet** | Number of days per week | |  |
|  | How many of the **last SEVEN DAYS** have you followed a healthful eating plan? | 0 1 2 3 4 5 6 7 | |  |
|  | **On average**, **over the past month**, how many DAYS PER WEEK have you followed your eating plan? | 0 1 2 3 4 5 6 7 | |  |
|  | On how many of the last SEVEN DAYS did you eat five or more servings of fruits and vegetables? | 0 1 2 3 4 5 6 7 | |  |
|  | On how many of the last SEVEN DAYS did you eat high fat foods such as red meat or full-fat dairy products? | 0 1 2 3 4 5 6 7 | |  |
|  | **Exercise** |  | |  |
|  | On how many of the last SEVEN DAYS did you participate in at least 30 minutes of physical activity? (Total minutes of continuous activity, including walking). | 0 1 2 3 4 5 6 7 | |  |
|  | On how many of the last SEVEN DAYS did you participate in a specific exercise session (such as swimming, walking, biking) other than what you do around the house or as part of your work? | 0 1 2 3 4 5 6 7 | |  |
|  | **Blood Sugar Testing** |  | | |
|  | On how many of the last SEVEN DAYS did you test your blood sugar? | 0 1 2 3 4 5 6 7 | |  |
|  | On how many of the last SEVEN DAYS did you test your blood sugar the number of times recommended by your health care provider? | 0 1 2 3 4 5 6 7 | |  |
|  | **Foot Care** |  | | |
|  | On how many of the last SEVEN DAYS did you inspect the inside of your shoes? | 0 1 2 3 4 5 6 7 | |  |
|  | On how many of the last SEVEN DAYS did you check your feet? | 0 1 2 3 4 5 6 7 | |  |

|  | **II. Behavioral Factors** | Response classification | | Code |
| --- | --- | --- | --- | --- |
|  | Have you ever smoked any tobacco products, such as cigarettes, cigars or pipes? | 1. Yes → If ‘Yes’ Go to 44  2. No → If ‘No’ Go to 50 | |  |
|  | Do you currently smoke any tobacco products, such as cigarettes, cigars or pipes? | 1. Yes  2. No | |  |
|  | Have you smoked a cigarette—even one  Puff—during the past SEVEN DAYS? | 1. Yes  2. No | |  |
|  | Have you ever smoked tobacco products daily? | 1. Yes  2. No | |  |
|  | How old were you when you **first started** smoking? | ___________ Age (years) | |  |
|  | On average, how many of the following products did you smoke each day/week? (IF LESS THAN DAILY, RECORD WEEKLY) | DAILY↓ | WEEKLY↓ |  |
|  | Manufactured cigarettes |  |  |  |
|  | Hand-rolled (local) cigarettes |  |  |  |
|  | Pipes („ Gaya ‟) full of tobacco |  |  |  |
|  | Number of Shisha sessions |  |  |  |
|  | Other ( specify) _________________ |  |  |  |
|  | How old were you when you stopped (if stopped) smoking? | ___________ Age (in years) | |  |
|  | Have you ever chewed chat? | 1. Yes 2. No **If ‘NO’ Go to 54** | |  |
|  | Do you currently chew chat? | 1. Yes 2. No | |  |
|  | Have you ever chewed chat daily? | 1. Yes 2. No | |  |
|  | In a typical month, How often did you chew chat? | 1. daily 2. 5-6 days per week 3. 1-4 days per week 4. 1-3 days per month 5. Less than once a month | |  |
|  | Have you ever consumed any alcohol such as beer,wine, spirits or [local drinks like Sewa, Miyes, Areqe,  Katikala]? | 1. Yes 2. No **If ‘NO’ Go to 59** | |  |
|  | During the past 12 months, how frequently have you had at least one standard alcoholic drink? | 1. Daily 2. 5-6 days per week 3. 3-4 days per week 4. 1-2 days per week 5. 1-3 days per month 6. Less than once a month 7. Never 7 | |  |
|  | When you drank alcohol, on average, how many standard alcoholic drinks did you have during one drinking occasion? | ________drinks | |  |
|  | What was the largest number of standard alcoholic drinks you had on a single occasion, counting all types of alcoholic drinks together? | Largest number _________ | |  |
|  | In a typical month, how many times did you have six or more standard drinks in a single drinking occasion? | ____________Number of times | |  |
|  | Have you ever used top added salt on your plate? | 1. Yes 2. No | |  |
|  | How often do you add salt or a salty sauce to your food right before you eat it or as you are eating it? | 1. Always 2. Often 3. Sometimes 4. Rarely | |  |
|  | Do you do any vigorous-intensity activities including work activity or sports, fitness or recreational (leisure) activities that cause large increases in breathing or heart rate like [carrying or lifting heavy loads, digging or construction work] or [running or football or lifting weight] for at least 10 minutes continuously? | 1. Yes → If ‘Yes’ Go to 62  2. No → If ‘No’ Go to 64 | |  |
|  | In a typical week, on how many days do you do vigorous-intensity work activities or sports, fitness or recreational (leisure) activities? | ___________days | |  |
|  | How much time do you spend doing vigorous-intensity work activities or sports, fitness or recreational activities on a typical day? | ____:_____Hr:min /day | |  |
|  | Do you do any moderate-intensity work activities or sports, fitness or recreational (leisure) activities that cause a small increase in breathing or heart rate such as brisk walking [or carrying light loads], [walking, cycling, swimming, volleyball] for at least 10 minutes continuously? | 1. Yes → If ‘Yes’ Go to 65  2. No → If ‘No’ Go to 67 | |  |
|  | In a typical week, on how many days do you do moderate-intensity work activities sports, fitness or recreational (leisure) activities? | _____________days | |  |
|  | How much time do you spend doing moderate-intensity work activities or sports, fitness or recreational (leisure) activities on a typical day? | ____:_____Hr:min/day | |  |
| **III. Physical Measurements and laboratory measurement** | | | | |
|  | Blood Pressure (measured twice) | 1. ________/________mmHg (systolic/diastolic) 2. ________/________mmHg (systolic/diastolic) | |  |
|  | Height | ___________cm | |  |
|  | Weight (kg) | ___________Kg | |  |
|  | Waist circumference | ___________cm | |  |
|  | Hip circumference | ___________cm | |  |
|  | Fasting blood sugar **(mg/dl)**  (four Consecutive measurements) | 1.__________ 2.___________ 3.__________ 4.__________ | |  |
|  | HbA1C | _____________ | |  |
|  | Serum triglyceride | _________________mg/dl | |  |
|  | Serum total cholesterol | _______________ mg/dl | |  |
|  | Serum HDL | _______________ mg/dl | |  |
|  | Serum LDL | _______________ mg/dl | |  |
|  | Serum creatinine (Baseline and recent) | 1.________mg/dl 2.________mg/dl | |  |
|  | Urine analysis (Albumin, glucose ,ketone ,blood)…(Baseline and recent) | 1.Alb___ gluc___ ket___ Bld ____  2.Alb___ gluc___ ket___ Bld ____ | |  |
| **Diabetic Complications** ** | | | | |
|  | Diabetic retinopathy | 1. Yes 2. No | |  |
|  | Diabetic neuropathy | 1. Yes 2. No | |  |
|  | Diabetic nephropathy | 1. Yes 2. No | |  |
|  | Cardiovascular diseases | 1. Yes 2. No | |  |
|  | Foot complication | 1. Yes 2. No | |  |
|  | Diabetic ketoacidosis (DKA) | 1. Yes 2. No | |  |
|  | Hyperosmolar hyperglycemic state(HHS) | 1. Yes 2. No | |  |
|  | Hypoglycemia | 1. Yes 2. No | |  |

Name of data collector _________________ Signature __________

Name of Supervisor __________________ Signature ___________
